# Prolyl hydroxylase domain 2 reduction enhances skeletal muscle tissue regeneration after soft tissue trauma in mice

**DOI:** 10.1371/journal.pone.0233261

**Published:** 2020-05-15

**Authors:** Stephan Settelmeier, Timm Schreiber, Joni Mäki, Nadiya Byts, Peppi Koivunen, Johanna Myllyharju, Joachim Fandrey, Sandra Winning

**Affiliations:** 1 Institut für Physiologie, Universität Duisburg-Essen, Essen, Germany; 2 Biocenter Oulu and Faculty of Biochemistry and Molecular Medicine, University of Oulu, Oulu, Finland; Duke University, UNITED STATES

## Abstract

The transcription factor Hypoxia-inducible factor 1 (HIF-1) plays a pivotal role in tissue regeneration. HIF-1 is negatively controlled by O_2_-dependent prolyl hydroxylases with a predominant role of prolyl hydroxylase 2 isoform (*Phd2)*. Transgenic mice, hypomorphic for this isoform, accumulate more HIF-1 under normoxic conditions. Using these mice, we investigated the influence of *Phd2* and HIF-1 on the regenerative capability of skeletal muscle tissue after myotrauma. *Phd2*-hypomorphic and wild type mice (on C57Bl/6 background) were grouped with regeneration times from 6 to 168 hours after closed mechanic muscle trauma to the hind limb. Tissue samples were analysed by immuno-staining and real-time PCR. Bone marrow derived macrophages of wild type and *Phd2*-hypomorphic mice were isolated and analysed via flow cytometry and quantitative real-time PCR. *Phd2* reduction led to a higher regenerative capability due to enhanced activation of myogenic factors accompanied by induction of genes responsible for glucose and lactate metabolism in *Phd2*-hypomorphic mice. Macrophage infiltration into the trauma areas in hypomorphic mice started earlier and was more pronounced compared to wild type mice. *Phd2*-hypomorphic mice also showed higher numbers of macrophages in areas with sustained trauma 72 hours after myotrauma application. In conclusion, we postulate that the HIF-1 pathway is activated secondary to a *Phd2* reduction which may lead to i) higher activation of myogenic factors, ii) increased number of positive stem cell proliferation markers, and iii) accelerated macrophage recruitment to areas of trauma, resulting in faster muscle tissue regeneration after myotrauma. With the current development of prolyl hydroxylase domain inhibitors, our findings point towards a potential clinical benefit after myotrauma.

## Introduction

Skeletal muscle tissue has a high regenerative capacity after myotrauma and assigns a pivotal role to Hypoxia-inducible factor 1 (HIF-1) in tissue regeneration [1]. In a murine excisional wound model with fibroblast-specific HIF-1 deletion, wound healing and neovascularization was impaired and showed significantly more tissue necrosis [2]. HIF-1α activation promotes myogenesis in vitro while HIF-1α inhibition leads to diminished myoblast differentiation [3]. In this study, we set out to investigate the impact of *Phd2* reduction and consecutive HIF-1 stabilization in tissue regeneration using a model of soft tissue trauma.

HIF-1, a key regulator in cellular oxygen homeostasis, is formed as a dimer complex of the oxygen labile HIF-1α and the constitutive HIF-1β subunit [4]. Oxygen lability of HIF-1α depends on the enzymatic activity of prolyl hydroxylases (PHDs) which requires oxygen [5–7]. Under normoxia, PHDs hydroxylate HIF-1α to initiate proteasomal degradation preventing dimerizing with HIF-1β into an active transcription factor complex. There are three isoforms of HIF prolyl hydroxylases (PHD1-3), with PHD2 showing highest oxygen-dependent activity. It therefore plays a dominant role as oxygen sensor [8]. In hypoxia, PHD activity and thus HIF-1α hydroxylation is reduced, HIF-1α becomes stable, accumulates and dimerizes to form the active HIF-1 transcription factor complex. HIF-1 then orchestrates the hypoxic response of the organism by inducing HIF-1 target genes to hypoxic conditions [6, 7, 9, 10].

PHD2 is of vital importance because knocking out *Phd2* in mouse embryos causes prenatal death. In contrast, hypomorphic *Phd2* mice with reduced PHD2 are viable. Reduction of *Phd2* protected hearts against acute ischemia-reperfusion injury [11–13], improved glucose and lipid metabolism and protected against the development of atherosclerosis [14, 15]. *Phd2* reduction stabilized HIF-1α under normoxia and protected skeletal muscle against ischemia-reperfusion injury [13].

Pathophysiologically, mechanical soft tissue trauma is characterized by ischemic and inflammatory hypoxia suggesting a role of HIF in myotrauma as well [16]. *In vitro*, skeletal muscle cells activated HIF-1 after exposure to skeletal muscle cell debris, as it is found after mechanical myotrauma [17]. *In vivo*, myotrauma in mice causes HIF-1 stabilisation after 24 hours and in particular, myeloid HIF-1α is essential for skeletal muscle regeneration [1]. In this model of mechanical soft tissue trauma, myeloid HIF-1α knockout significantly delayed myoblast proliferation and growth of regenerating muscle fibers [1].

Mononuclear satellite cells serve as the regenerative stem cell population for skeletal muscle in situations of overstraining and injury [18]. The paired box transcription factor Pax7 is a specific intracellular marker and is constantly expressed in adult satellite cells [19–21]. Pax7-positive satellite cells are mainly responsible for adult skeletal muscle regeneration and have the ability of self-renewal [22, 23]. Furthermore, Pax7-positive cells support the proliferation of progenitor cells while preventing them from premature differentiation and apoptosis [24]. Furthermore, myogenic regulatory factors (MRF) are expressed by proliferating muscle cells during specific differentiation states [19, 25]. HIF-1 is able to activate the expression of Pax7 through direct interaction with the Pax7 promoter [26], MyoD and myogenin are targets of hypoxia likewise [27].

In summary, at the beginning of regeneration, Pax7-expressing quiescent satellite cells migrate to the trauma zone, induce myogenic regulatory factors such as MyoD and start to proliferate. While differentiating into myoblasts, Pax7 expression decreases whereas MyoD and Myogenin expression increases [19, 20, 24, 28].

### Role of macrophages in skeletal muscle regeneration

The central role of macrophages in skeletal muscle regeneration is well described [29]. In a bipolar model, macrophages polarise either into an early proinflammatory phenotype or later into an inflammatory response dampening and muscle cell regeneration promoting phenotype [19, 30]. Macrophage depletion impaired skeletal muscle regeneration in a contusion model, which showed a significant correlation between macrophages and myogenic markers [31]. Myeloid (= macrophage) knockout of HIF-1α led to delayed macrophage migration to a skeletal muscle injury site and an overall impaired tissue regeneration [1]. This study set out to determine the impact of *Phd*2-deficiency in skeletal muscle regeneration after soft tissue muscle trauma.

## Methods

### Ethical approval

All animal experiments were performed in accordance to the ethical guideline of the Finnish National Animal Experiment Board and approved by the Finnish National Animal Experiment Board (Eläinkoelautakunta, ELLA) (ESAVI/6154/04.10.07/2014). Animal experiments were performed at Biocenter Oulu and Faculty of Biochemistry and Molecular Medicine, University of Oulu, Finland.

### Hypomorphic mice & procedure of myotrauma

*Phd2*-hypomorphic mice used in this study were described previously [12]. These mice show decreased *Phd2* mRNA expression in skeletal muscle tissue and normoxic HIF-1α and HIF-2α stabilization as described [12, 13]. The mice were kept under standardized conditions in a specific-pathogen-free environment with a constant day and night rhythm, water and feeding *ad libitum*. The mice were healthy and fertile. Only male mice of matched age (mean: 12 ± 2 weeks) were used in all experiments to ensure a similar size and volume of the traumatized muscle. Average mean weight of mice was 27.08 ± 3,26 g. 62 mice were used *in toto* and were randomly grouped with a group size of 4–6 per condition.

### Procedure of myotrauma

For reproducible induction of a closed myotrauma, we used a drop-mass gadget designed by Crisco et al. and modified by Kerkweg et al. [32, 33]. A 69.2 g metal weight was dropped in free fall from a height of 51.6 cm onto a metal piston, which was placed on the hind limb muscle with a kinetic energy of 0.35 J resulting in a soft tissue trauma only without bone fractures [1]. Mice were anesthetized with ketamine/xylazine (0.01 mg (g BW)^-1^ i.p. with ketamine 50 mg (ml)^-1^ and xylazine 20 mg (ml)^-1^; Richter-Pharma, Wels, Austria) in the morning and treated with buprenorphine (0.01 mg (g BW)^-1^; CP-Pharma, Burgdorf, Germany) s.c. as an analgesic every eight hours. Atipamezol (0.02 mg (g BW)^-1^; CP-Pharma, Burgdorf, Germany) was used for the reversal of sedative and analgesic effects. Directly after procedure, mice were retransferred into their home cage. Two mice were sacrificed by cervical dislocation because of limping and not included in the analysis.

#### Muscle tissue sampling

At six, 24, 72, 96 and 168 hours after trauma induction, mice were sacrificed by cervical dislocation and bilaterally traumatized gastrocnemius muscles were harvested and then stored either in 4% paraformaldehyde or liquid nitrogen for later analysis. At every time point, sham animals served as control.

### RNA preparation and RT-PCR

RNA was isolated using the phenol/chloroform method [34]. Tissues were homogenized in guanidinium thiocyanate and RNA was subsequently extracted. The RNA concentration was measured photometrically. A total of 5 μg of RNA was converted into cDNA by using oligo-dT-nucleotides as the reverse transcriptase primer (Invitrogen/Life Technologies, Waltham, USA). Real-time quantification was done via a two-step PCR with denaturation steps at 95°C for 10 minutes and then 40 cycles at 95°C for 15 seconds and at 60°C for 90 seconds [1]. Ribosomal protein served as internal control. We analysed the results using the cycle-of-threshold-method [35]. Please refer to Table 1 for primers.

**Table 1:** List of primers and sequences.

Name of primer	Sequence
CCL2	5’ GTGCTGAAGACCTTAGGGCA 3’ AGCTGTAGTTTTTGTCACCAAGC
CCR2	5‘ TAGAGTGGAGGCAGGATCCAA 3‘ ACCTCAGTTCATCCACGGC
COX2	5‘TCCTCCTGGAACATGGACTC 3‘ CCCCAAAGATAGCATCTGGA
CXCR4	5‘ TGGAACCGATCAGTGTGAGT 3‘ TTGCCGACTATGCCAGTCAA
GLUT3	5‘ ATGGGGACAACGAAGGTGA 3‘ GCCAATCATGCCACCAACAG
HGF	5‘ CCTGTGCCTTGACTTAGCGA 3‘ GCCGGGCTGAAAGAATCAAAG
IGF1	5‘ ACGGCATTGTGGATGAGTG 3‘ TATGGCCTTCTGTCCAGGTC
MCT3	5‘ TGGTGAACTACGCCAAGGAC 3‘ AGGCAATGCAGAAAGCAACG
PFKM	5‘ GGAGAGCTAAAACTACAAGAGTGGA 3‘ CGCCCGTGAAGATACCAACT
*Phd2* (E1-E2)	5‘ CTGGGCAACTACAGGATAAAC 3‘ GCGTCCCAGTCTTTATTTAGATA
SDF1	5‘ CAGCCGTGCAACAATCTGAAG 3‘ CTGCATCAGTGACGGTAAACC
VEGF	5‘ CGTCCTGTGTGCCGCTGATGC 3‘ ACAAGGCTCACAGTGATTTTCTTGC

### Immunohistochemistry

We used paraformaldehyde-fixed, paraffin-embedded samples of one microgram tissue thickness as previously described [36]. In essence, heat-induced epitope retrieval was performed in a citrate buffer at pH 6.0 followed by diverse blocking steps and detection with the ABC Vectastain kit (Maravai Life Sciences, San Diego, USA). Immunohistochemistry for HIF-1α was done with the Dako CSAII-Kit (Dako Agilent, Santa Clara, USA). As primary antibodies we used rabbit-anti-HIF-1α (Cayman Chemical, Ann Arbor, USA Cat# 10006421, RRID:AB_409037) at a dilution of 1:10.000, rat-anti-F4/80 (Bio-Rad, Hercules, USA Cat# MCA497, RRID:AB_2098196) at a dilution of 1:200, rat-anti-VEGF (BioLegend, San Diego, USA Cat# 512901, RRID:AB_2212504) at a dilution of 1:300, rabbit-anti-PHD2 (novus Biologicals, Cat# 100–137, RRID:AB_350074 at a dilution of 1:200. For the detection of F4/80 and VEGF, biotinylated anti-rat antibody (Santa Cruz Biotechnology, Dallas, USA Cat# sc-2041, RRID:AB_631752) and for the detection of PHD2, biotinylated anti-rat antibody (Santa Cruz Biotechnology, Dallas, USA, Cat# sc-2040, RRID:AB_631743) was used as the secondary antibody (1:200).

### Immunofluorescent staining

We used paraformaldehyde-fixed, paraffin-embedded samples of one microgram tissue thickness. As primary antibodies, rabbit-anti-Pax7 (Abcam, Cambridge, UK, Cat# ab187339, RRID:AB_2813893) at a dilution of 1:200, mouse-anti-MyoD (Santa Cruz Biotechnology, Dallas, USA CAT# sc-377460, RRID:AB_2813894) at a dilution of 1:250 and mouse-anti-Myogenin (Santa Cruz Biotechnology, Dallas, USA Cat# sc-52903, RRID:AB_784707) at a dilution of 1:250. The signal was visualized by goat-anti-rabbit Alexa Fluor 568 and goat-anti-mouse Alexa Fluor 568 respectively (Invitrogen/Life Technologies, Waltham, USA, Cat# A-11004, RRID:AB_2534072 and Cat# A-11011, RRID:AB_143157).

### Quantitative analysis of histological markers

For quantitative analysis of histological markers, entire H&E-stained midbelly midline sections of the gastrocnemius muscle were digitalized, and four representative traumatized areas were exported as TIFF images and analysed in the ImageJ 1.50e based open-source program Fiji (RRID:SCR_002285) in a blind manner. At least 100 muscle fibers per image were integrated into the analysis. We identified the traumatic area, divided it into four random sections (two central traumatic sections and two in the border zone) and counted the total amount of myofibers and the total amount of centrally nucleated fibers. By means of morphologic criteria, we measured the proportions of traumatized muscle fibers to total muscle fibers and regenerating muscle fibers to total muscle fibers. Regenerating myofibers were defined as centrally nucleated myofibers. For quantitative analysis of immunofluorescent markers, four representative traumatized areas were exported as TIFF images, color channels were fused and analysed by counting muscle cells in the ImageJ 1.50e based open-source program Fiji in a blind manner. For analysis of immunohistochemical images, we identified regions with a high proportion of regenerating muscle cells to asses protein expression as cell-specifically as possible. We then analysed binary images: DAB-positive cells were determined and digitally counted using the IHC-toolbox in the ImageJ 1.50e based open-source program Fiji in a blinded manner.

### BMDM analysis/macrophages

Bone marrow derived macrophages were isolated and cultured as previously described [37]. Analysis was performed seven days after extraction from the bone marrow. The mRNA extraction technique has been described above. For flow cytometry, 2∙10^6^ macrophages were incubated with Fc block (dilution of 1:200; Biolegend, Koblenz, Germany, Cat# 101319, RRID:AB_1574973) and F4/80-FITC (dilution of 1:400; clone BM8, Biolegend, Koblenz, Germany, Cat# 123120, RRID:AB_893479) and CD206-APC antibody (dilution of 1:200; clone C068C2, Biolegend, Koblenz, Germany, Cat# 141707, RRID:AB_10896057) as well as eBioscience Fixable Viability Dye eFluor 780 (referred to as FVD; used according to manufacturer´s instructions, ThermoFisher, Darmstadt, Germany, Cat# 65-0865-18) and then analysed with a Canto II cytometer (Becton Dickinson). Therefore, only FVD-negative cells were included in the analysis. Cells were gated for F4/80 positivity. Given numbers show F4/80 positive cells that are also positive for CD206.

### Statistics

All data were analysed using GraphPad Prism 5 software (GraphPad Software Inc., San Diego, USA, RRID:SCR_002798). The statistical significance of differences was calculated by Two-way ANOVA and post-hoc multiple comparison of Sidak. The statistical significance of differences of FACS analysis of BMDM was calculated by the Student´s t-test.

## Results

### *Phd2*-hypomorphic mice show a higher regenerating proportion of muscle fibers

**Fig 1:**
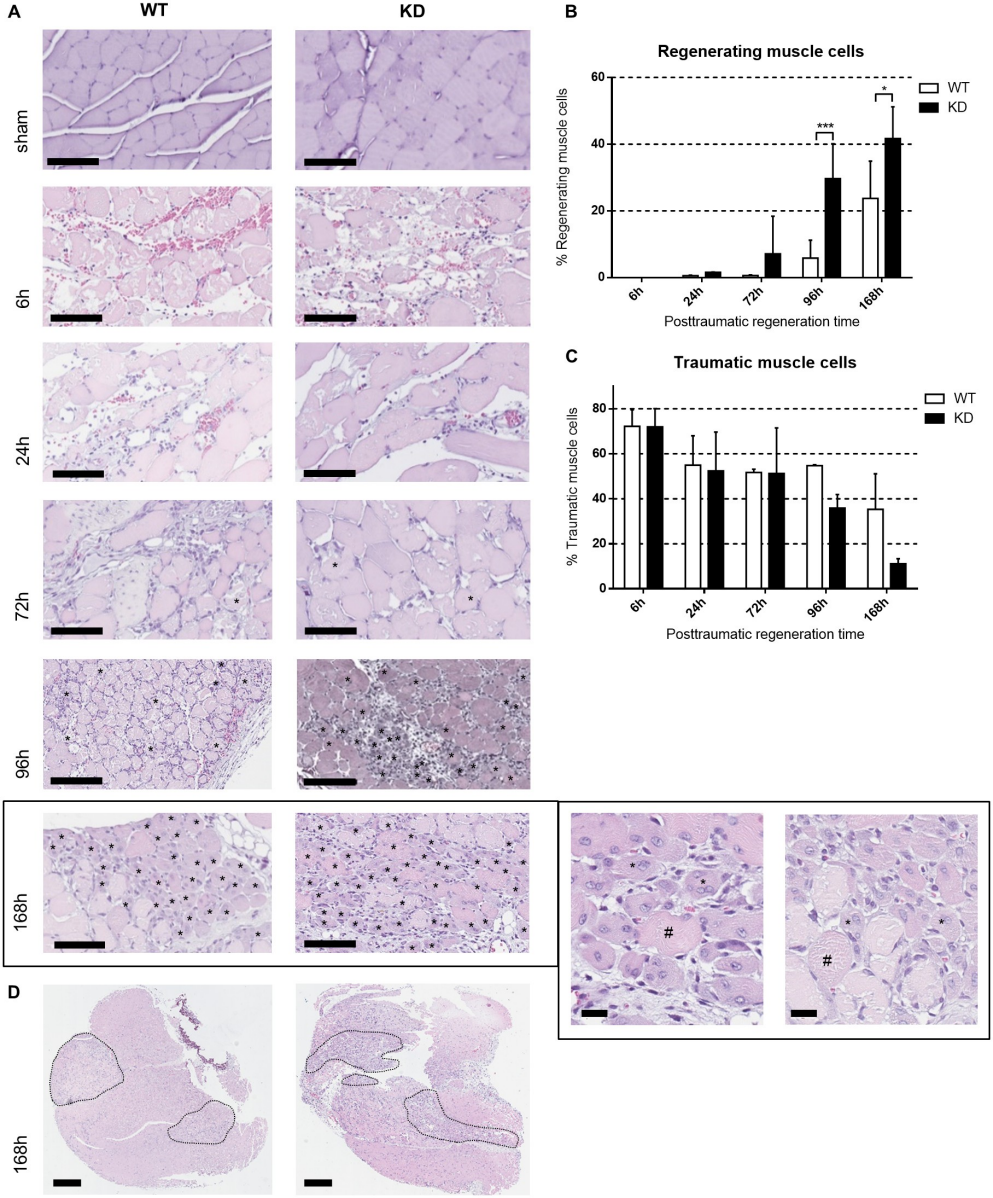
Myotrauma is resolved faster in *Phd2*-hypomorphic mice. (A) H&E staining of skeletal muscle at indicated time points after myotrauma (6 h, 24 h, 72 h, 96 h und 168 h) and of sham-treated wild type (WT) and *Phd2*-hypomorphic (knockdown, KD) mice. Loss of muscle fiber architecture, haemorrhage and vacuole formation occur early after trauma and are followed by increased immune cell invasion and tissue reorganisation. Regenerating muscle cells are characterised by central nuclei and marked with asterisks (*). Shown are slides of 1 μm thickness (200x magnification; scale bar: 200 μm). Magnification image of 168h: regenerating (*) and non-regenerating (#) muscle cells are displayed exemplarily. Due to the magnification of central trauma areas, genotype differences may not be visible in these images (scale bar: 20μm). (B) Relative numbers of regenerating muscle cells (in % of all intact muscle cells) are significantly increased in KD animals compared to WT; n = 4–6. (C) Relative numbers of traumatic muscle cells (in % of all muscle cells) decrease faster in KD mice compared to WT; n = 4–6. * P < 0.05, *** P < 0.001. (D) Representative images of traumatized muscles in full section. Indicated are traumatic areas. Due to contusion artefacts, areas may appear separated. We observed no difference in acute trauma size between the genotypes indicating a standardized impact procedure. (scale bar: 500μm).

We conducted experiments to determine whether *Phd2* reduction and the subsequent normoxic HIF stabilization made a phenotypic difference during muscle trauma regeneration. Using hematoxylin and eosin staining, we identified regenerating muscle fibers with central nuclei and determined the ratio of regenerating to the total number of muscle fibers. We detected a significantly higher proportion of regenerating muscle fibers in *Phd2*-hypomorphic mice (indicated in figures as “KD”) 96 and 168 hours after trauma induction (Fig 1B). Loss of muscle fiber architecture, hemorrhage and vacuole formation occurred early after trauma and were followed by an increased rate of immune cell invasion and tissue reorganization (Fig 1A). The number of traumatic muscle cells did not differ at early time points after myotrauma but seemed lower in *Phd2*-hypomorphic mice 168 hours after myotrauma due to higher fiber regeneration (Fig 1C). We observed no difference in acute trauma size between the genotypes indicating a standardized impact procedure (Fig 1D).

#### *Phd2-*hypomorphic mice show more proliferation marker positive myocytes after trauma

**Fig 2:**
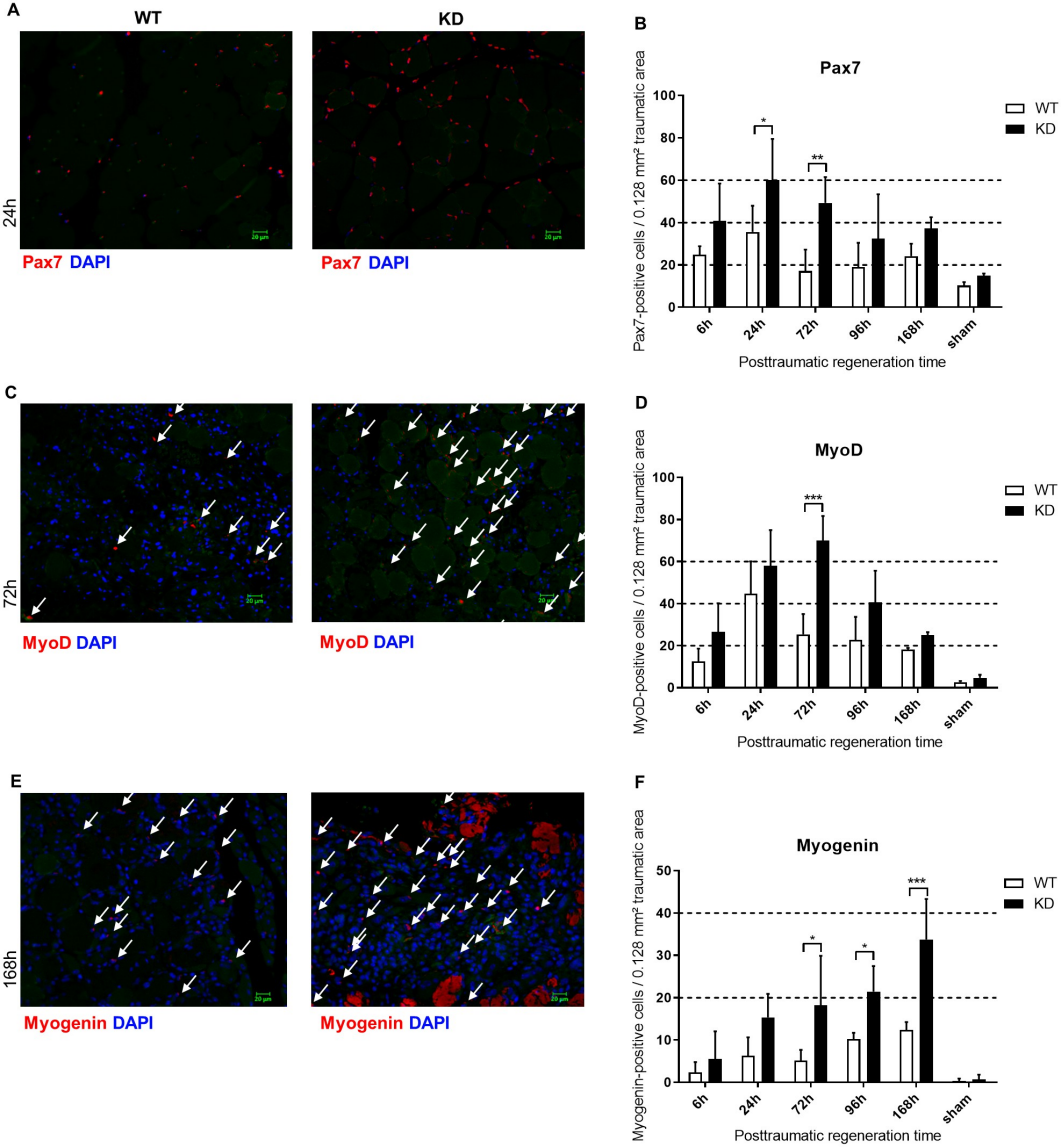
Skeletal muscle repair is enhanced in *Phd2*-hypomorphic mice. (A/C/E) Immunofluorescence staining of Pax7, MyoD and myogenin in skeletal muscle of traumatic and sham treated wild type (WT) and *Phd2*-hypomorphic (KD) mice (time periods after trauma application are indicated). Stained are Pax7 (red), nuclei (DAPI; blue), intrinsic fluorescence (green). Colours have been adjusted for visualization in a linear fashion. The myogenin signals in (E) are cellular signals, red-stained myofibers are artificial due to technical reasons. Shown are slides of 1 μm thickness (200x magnification; scale bar: 20 μm). (B/D/F) Pax7-, MyoD-, and myogenin-positive nuclei per 0.128 mm^2^ area of trauma were counted from the stained slides. Time courses of induction of Pax7, MyoD and myogenin reveal subsequent activation of the named factors of the myogenic program. Inductions are significantly higher in *Phd2*-hypomorphic mice. Mean ± SD; n 4–6; sham: n = 3. * P < 0.05, ** P < 0.01, *** P < 0.001.

To further analyse the observed higher regenerative potential of *Phd2*-hypomorphic mice, we investigated the number of Pax7-positive satellite cells after immunofluorescent staining (Fig 2A). We observed a significantly higher induction of Pax7-positive cells in *Phd2*-hypomorphic mice compared to wild type mice 24 hours and 72 hours after myotrauma, whereas there was no difference between the groups in the sham treated mice (Fig 2B). During regeneration, activated satellite cells and myoblasts express the transcription factor MyoD. Using immunofluorescent staining, we investigated MyoD-expressing myocytes (Fig 2C). Compared to the sham group, we observed an increase in MyoD-positive myocytes six hours after myotrauma. 72 hours after myotrauma, the extent of MyoD expression decreased in wild type mice while at the same time point, *Phd2*-hypomorphic mice showed about three times as many MyoD-positive myocytes. 96 hours after the induced trauma, *Phd2*-hypomorphic mice still exhibited a higher degree of MyoD positivity compared to the wild type mice (Fig 2D).

During the myogenic program, regenerating muscle cells express the transcription factor myogenin. Using immunofluorescent staining, we investigated myogenin-expressing myocytes (Fig 2E). Animals from the sham groups showed nearly no myogenin positivity. Compared to wild type animals, the number of myogenin-positive cells was significantly increased at 72, 96 and 168 hours after myotrauma in *Phd2*-hypomorphic mice (Fig 2F).

#### HIF-1α accumulation & *Phd2* deficiency in myotrauma

**Fig 3:**
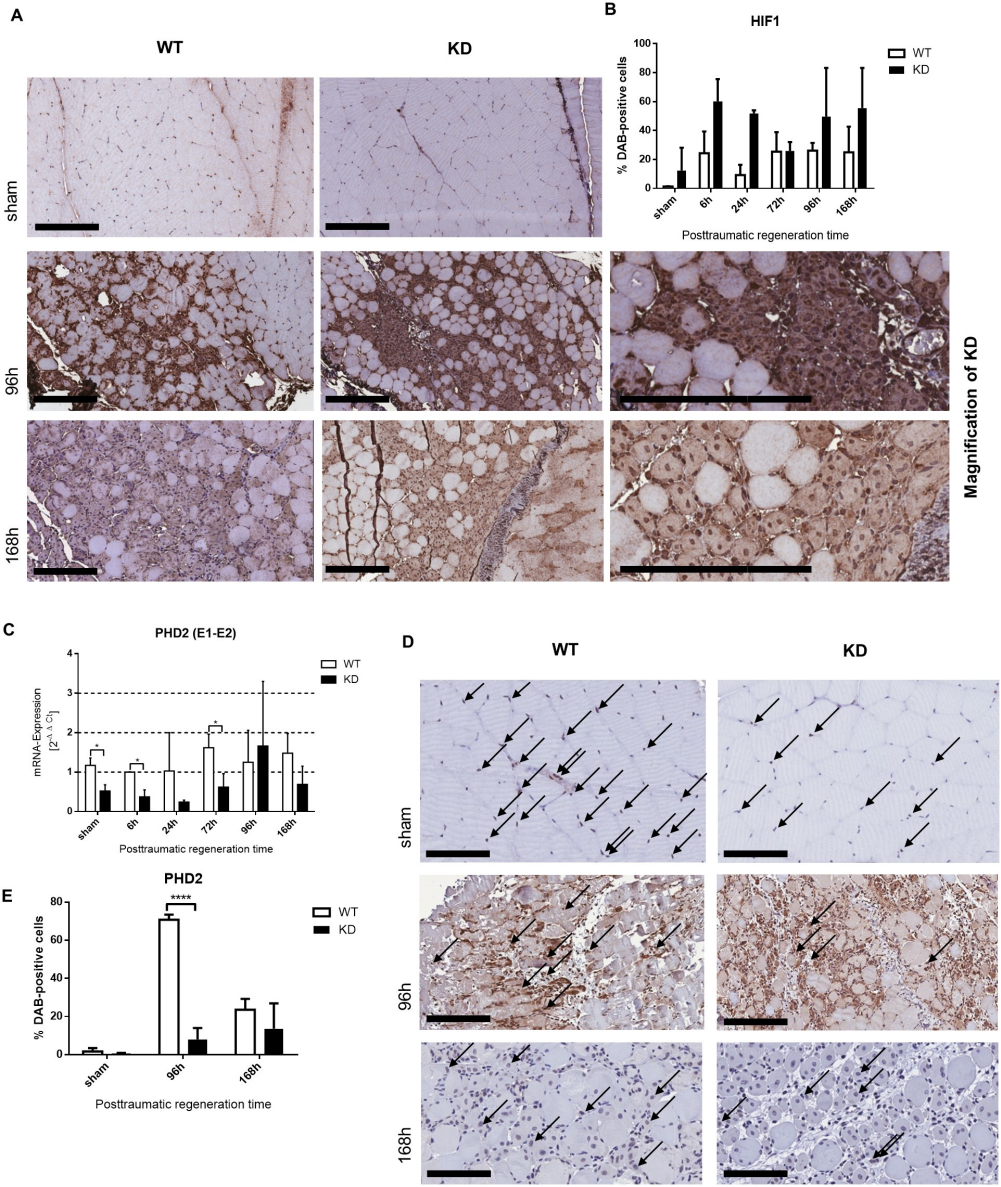
HIF-1α protein stabilization in myotrauma is comparable between WT and KD. (A) Immunohistochemical staining of HIF-1α in skeletal muscle of traumatic and sham treated wild type (WT) and *Phd2*-hypomorphic (KD) mice. Cell nuclei positive for HIF-1α at early time points are restricted to invaded immune cells. Over time there are increasing numbers of HIF-1α positive myonuclei. Shown are slides of 1 μm thickness (200x magnification; 600x magnification; scale bar: 200 μm). (B) Quantification of presented immunohistochemical staining (% of DAB-positive cells) of regenerating muscle cells. (D) Immunohistochemical staining of PHD2 in skeletal muscle of traumatic and sham treated wildtype (WT) and *Phd2*-hypomorphic (KD) mice. Seven days after myotrauma, *Phd2*-hypomorphic mice exhibited reduced numbers of positively stained myonuclei in representative regenerating areas compared to wild type mice (arrows). Shown are slides of 1 μm thickness (200x magnification; scale bar: 200 μm). (C) qPCR analysis (whole muscle tissue) of *Phd2* Exon 1 (E1)—Exon 2 (E2) for knockdown control (E) analysis of DAB-positive cells in areas containing almost exclusively regenerating muscle cells revealed lower PHD2 staining; mean ± SD; n = 4–8; sham: n = 3. * P < 0.05.

Immunohistochemical staining of HIF-1α in the experimental and sham *Phd2*-hypomorphic mice and wild type mice demonstrated HIF-1α positivity restricted to invading immune cells six to 72 hours after trauma. After 72 hours, increasing numbers of HIF-1α-positive myonuclei were detectable (Fig 3A). We were not able to observe any obvious differences in the expression intensity of nuclear HIF-1α between wild type and *Phd2*-hypomorphic mice, nevertheless, in the representative images, DAB-positive cells for HIF-1α in *Phd2*-hypomorphic mice were more numerous. We observed significantly lower *Phd2* mRNA expression compared to wild type mice in whole muscle tissue lysates at 6 and 72 hours after trauma but also in sham treated wild type compared to *Phd2*-hypomorphic mice (Fig 3C). Immunohistochemical staining of PHD2 showed reduced PHD2 expression in skeletal muscle nuclei of sham *Phd2*-hypomorphic mice (Fig 3D). 96 hours and 168h after myotrauma, *Phd2*-hypomorphic mice exhibited reduced numbers of positively myonuclei stained for *Phd2* in representative regenerating areas compared to wild type mice (see arrows in Fig 3D and 3E).

#### Elevated levels of vascular endothelial growth factor and key growth factors in myotrauma regeneration

**Fig 4:**
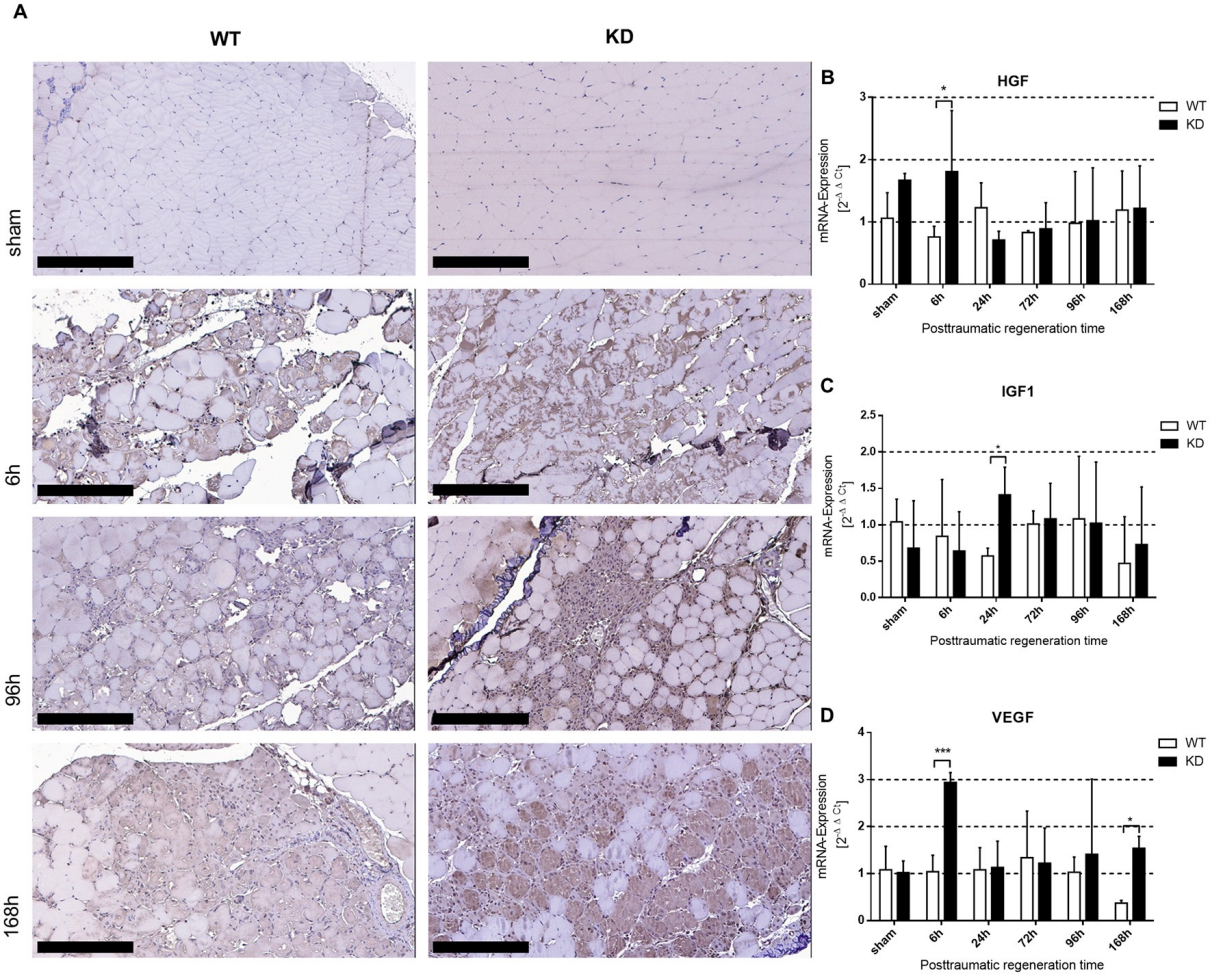
VEGF expression after myotrauma is enhanced in *Phd2*-hypomorphic mice. (A) Immunohistochemical staining of VEGF of traumatic and sham treated wild type (WT) and *Phd2*-hypomorphic (KD) mice. Early after trauma, VEGF staining can be detected in areas of invaded immune cells. At all time points staining is much more intensive in KD mice compared to WT. Shown are slides of 1 μm thickness (200x magnification; scale bar: 200 μm). (B-D) qPCR analysis (whole muscle tissue) of key growth factors in myotrauma (B) hepatocyte growth factor, *Hgf*; (C) insulin-like growth factor, *Igf1*; (D) vascular-endothelial growth factor, *Vegf*. *Hgf* and *Vegf* expression are significantly induced in KD mice 6h after myotrauma. Significant induction of *Igf1* expression in KD mice 24 h after trauma (mean ± SD; n = 4–6; sham: n = 3). * P < 0.05, *** P < 0.001.

We stained for vascular endothelial growth factor (VEGF) as a HIF-1 dependent gene with immunohistochemistry. We detected VEGF staining 6 to 24 hours after trauma in areas where immune cells had invaded. Beginning at 96 hours after trauma induction, we also observed VEGF-positivity in regenerating muscle fibers. The degree of staining was much more intense in *Phd2*-hypomorphic mice compared to wild type at any observed time point (Fig 4A). Key growth factors in myotrauma were studied using qPCR. *HGF* and *VEGF* expression were significantly higher in *Phd2*-hypomorphic mice 6 hours after myotrauma; *IGF1* was significantly increased in *Phd2*-hypomorphic mice 24 hours after myotrauma (Fig 4C).

#### Elevated levels of chemokine and cytokine expression in skeletal muscle trauma

**Fig 5:**
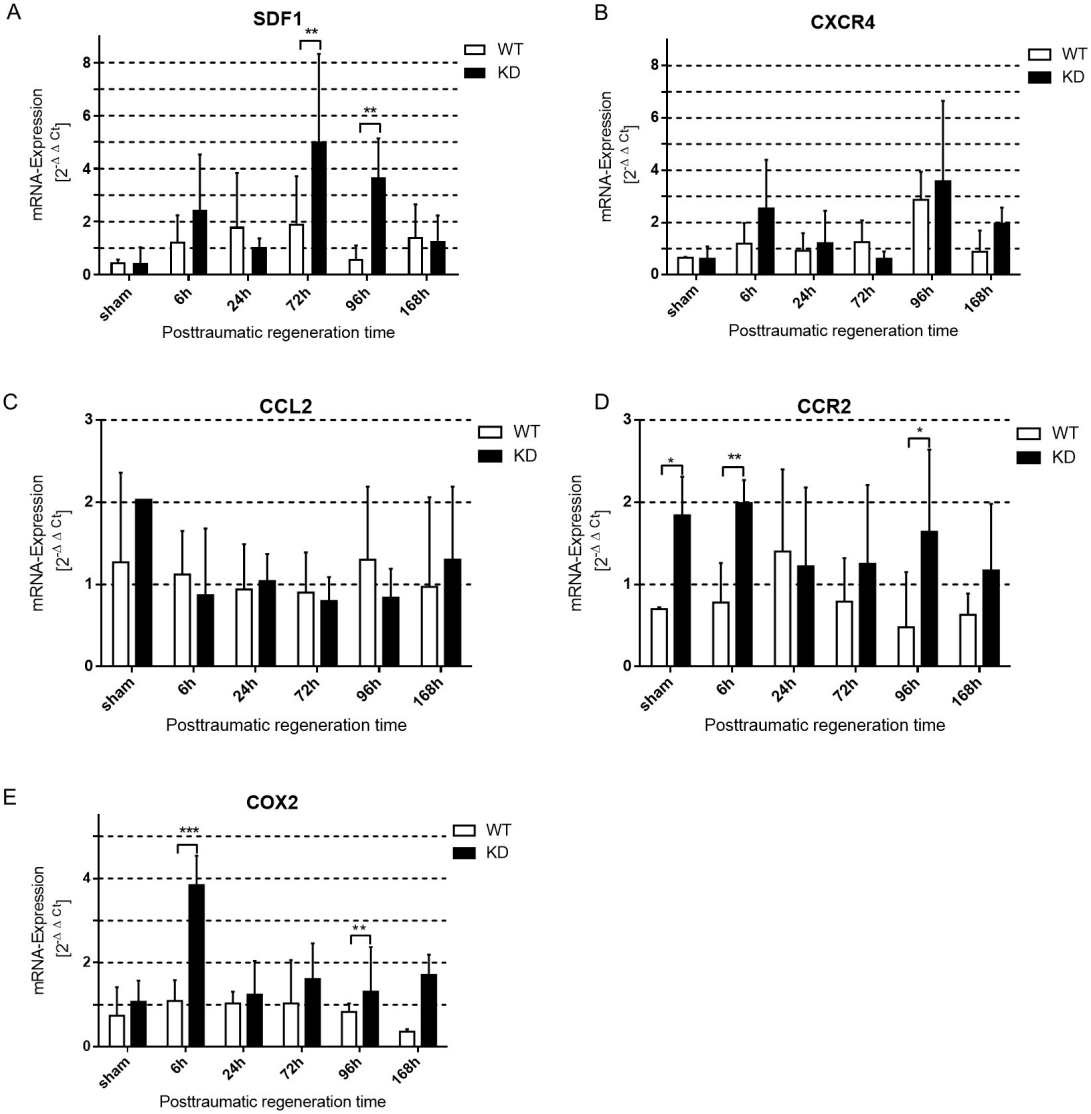
Chemokine/Cytokine induction in skeletal muscle trauma is higher in *Phd2*-hypomorphic mice. qPCR analysis (whole muscle tissue) of selected chemokines and cytokines in myotrauma of traumatic and sham treated wild type (WT) and *Phd2*-hypomorphic (KD) mice. (A) stroma cell-derived factor 1, *Sdf1*; (B) CXC-motive chemokine receptor 4, *Cxcr4*; (C) C-C ligand 2, *Ccl2*; (D) chemokine receptor 2, *Ccr2*; (E) cyclooxygenase 2, *Cox2*). *Sdf1* expression is significantly induced in KD mice at later time points (72 and 96 hours after myotrauma), *Ccr2* expression is significantly induced in sham treated mice and 6 h and 96 h, whereas *Cox2* expression is significantly induced in KD mice 6 h and 168 h after myotrauma (mean ± SD; n 4–6; sham: n = 3). * P < 0.05, ** P < 0.01, *** P < 0.001.

SDF1 and its receptor CXCR4 are important in recruiting myogenic cells and in wound healing. Six hours after trauma induction, we observed increased levels of *Sdf1* and *Cxcr4* mRNA in both genotypes. The time course showed significantly higher levels of the ligand *Sdf1* 72 hours and 96 hours after trauma in *Phd2*-hypomorphic mice, while there was no significant difference in *Cxcr4*-expression. We only observed an increase in both genotypes 96 hours after trauma (Fig 5A and 5B). Skeletal muscle regeneration is linked to the activity of immune cells within the traumatic area. CCL2 and its receptor CCR2 control the recruitment and activation of macrophages. Ccr2 mRNA expression was significantly increased in Phd2-hypomorphic mice at six and 96 hours after trauma induction (Fig 5C and 5D). COX2 is responsible for the synthesis of prostaglandins in the early stages of mice myotrauma regeneration [38]. It is expressed by neutrophils and macrophages [39]. Cox2 mRNA expression of whole tissue lysate was significantly increased in Phd2-hypomorphic mice at six and 168 hours after myotrauma induction (Fig 5E).

#### Macrophages in skeletal muscle trauma

**Fig 6:**
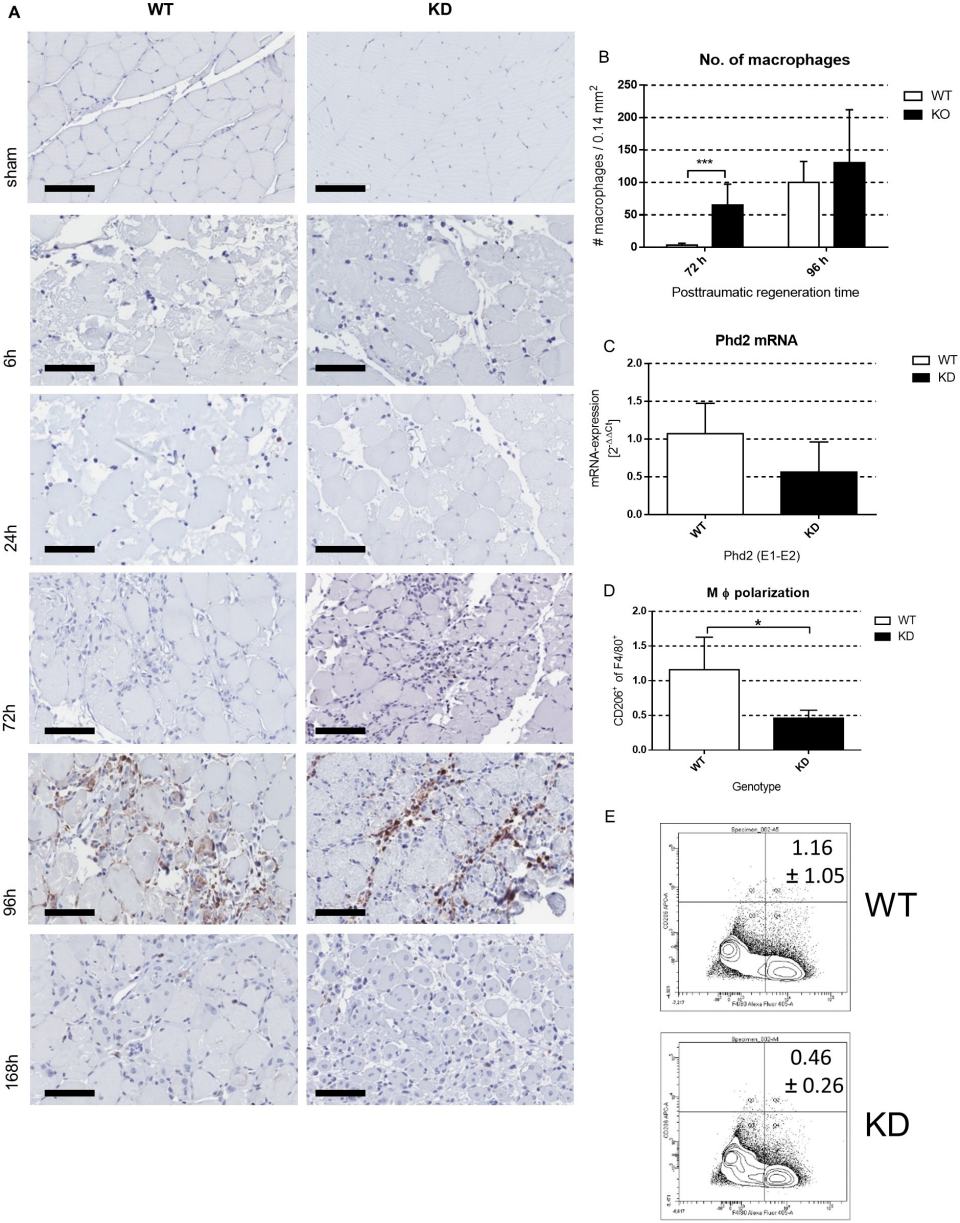
Macrophage recruitment to traumatic areas starts earlier in *Phd2*-hypomorphic mice. (A) Immunohistochemical staining of F4/80 of traumatic and sham treated wild type (WT) and *Phd2*-hypomorphic (KD) mice. Shown are slides of 1 μm thickness (200x magnification; scale bar: 200 μm). 72 hours after trauma, macrophage invasion is significantly higher in KD animals (B). (C) Bone marrow derived macrophages of KD mice show a 50% reduction in *Phd2* Exon 1 (E1)—Exon 2 (E2) in qPCR analysis (mean ± SD; n = 5). (D) Flow cytometric analysis of bone marrow derived macrophages of WT and KD cells reveals a significantly reduced expression of the surface marker CD206, which characterizes anti-inflammatory macrophages, in KD cells (mean ± SD; n = 5). (E) Gating strategy of F4/80^+^CD206^+^ bone marrow derived macrophages from WT and KD animals. Mean percentages (± SD; n = 5) of this population are indicated in the upper right corner * P < 0.05, *** P < 0.001.

Immunohistochemistry staining for F4/80 was used to stain activated macrophages in myotrauma. Macrophages started to infiltrate areas of muscle trauma in *Phd2*-hypomorphic mice 72 hours after trauma and were significantly higher in number in the trauma area compared to wild type mice (Fig 6A and 6B). This might be facilitated by a significantly increased mRNA expression of the matrix metallopeptidase 9 in *Phd2*-hypomorphic mice at this time point (S1H Fig). In wild type mice, macrophages were not detected earlier than 96 hours after trauma induction at the trauma sites. The number of macrophages detected at this time point was not different between wild type and *Phd2*-hypomorphic animals.

Interestingly, isolated bone marrow derived macrophages (BMDM) from wild type and *Phd2*-hypomorphic mice did not show significant differences in the expression of *Phd2* mRNA, indicating that the knockdown of *Phd2* might only mildly affect macrophages (Fig 6C). Nonetheless, FACS analysis of non-stimulated BMDM from wild type and *Phd2*-hypomorphic mice revealed a significantly higher expression of CD206, a classic marker for anti-inflammatory macrophages in the wild type cells (Fig 6D).

#### Genes of the glucose and lactate metabolism after trauma are enhanced in Phd2-hypomorphic mice

**Fig 7:**
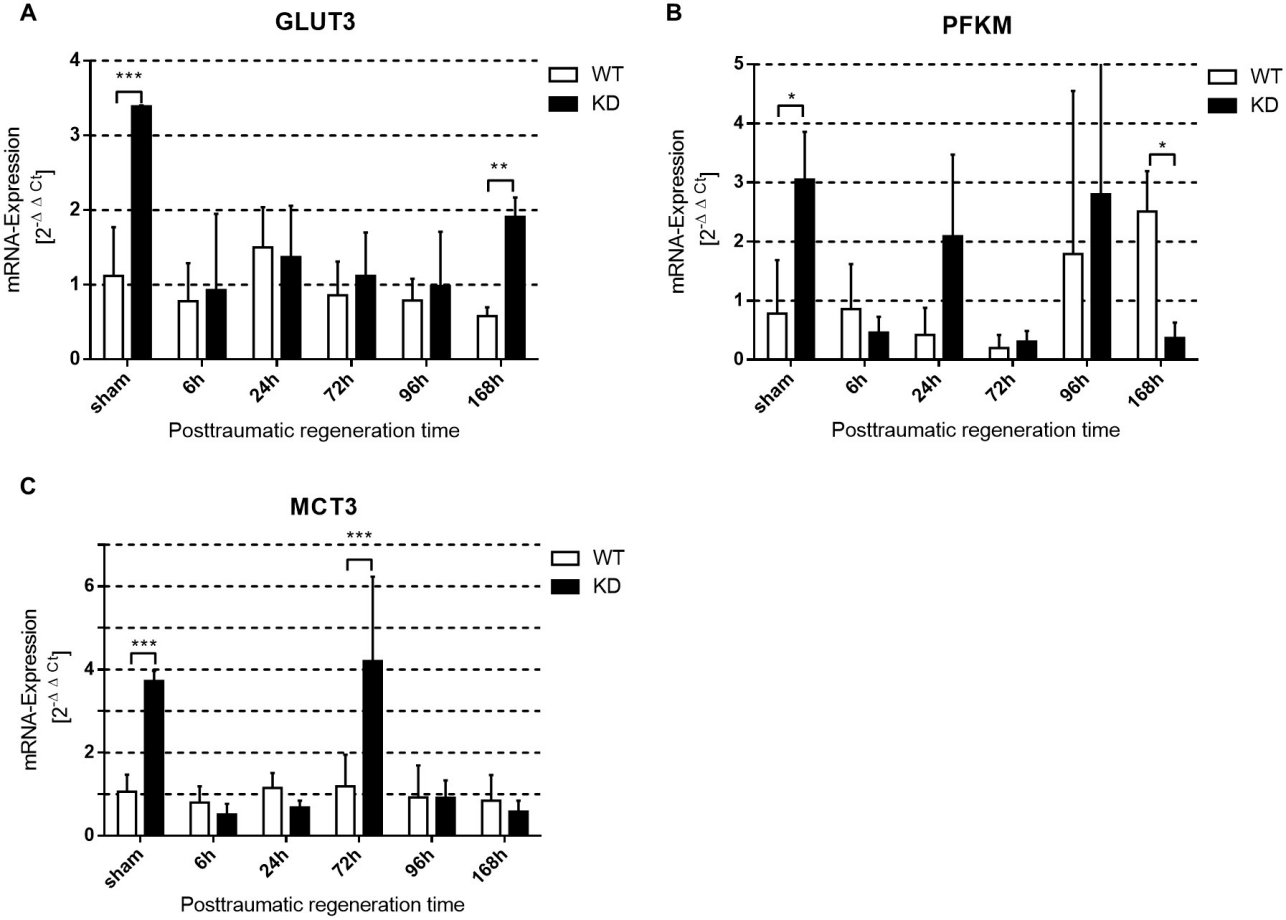
Genes of the glucose and lactate metabolism after trauma are enhanced in *Phd2*-hypomorphic mice. qPCR analysis (whole muscle tissue) of selected factors of glucose and lactate metabolism in myotrauma of traumatic and sham treated wild type (WT) and Phd2-hypomorphic (KD) mice. (A) glucose transporter 3, *Glut3*; (B) phosphofructokinase muscle type, *Pfkm*; (C) monocarboxylate transporter 3; *Mct3*). *Glut3* is significantly induced in SHAM KD mice and 168h after trauma. There is a significant induction of *Pfkm* 168h after trauma in WT mice while *Pfkm* expression is reduced in KD mice. We observed a significantly induced *Mct3* expression in sham treated KD mice and 72h after myotrauma (mean ± SD; n = 4–6; sham: n = 3). * P < 0.05, ** P < 0.01, *** P < 0.001.

qPCR analysis of whole tissue lysate revealed a more pronounced expression of genes for glucose and lactate metabolism in *Phd2*-hypomorphic mice. *Glut3* mRNA was significantly induced in *Phd2*-hypomorphic mice 168 hours after myotrauma but also in sham treated mice (Fig 7A). *Pfkm* was significantly higher in sham treated *Phd2*-hypomorphic mice but then significantly higher in wild type mice 168 hours after myotrauma (Fig 7B). We observed a significant induction of *Mct3* expression in *Phd2*-hypomorphic sham treated mice and 72 hours after myotrauma (Fig 7C).

## Discussion & conclusion

In this study, we investigated the impact of *Phd2* reduction on skeletal muscle injury and repair in a model of soft tissue trauma. Taking our given results into account, we hypothesize that *Phd2* reduction leads to enhanced skeletal muscle regeneration due to HIF-1-mediated effects.

After induction of myotrauma to the mouse hind limb, we observed no difference in acute trauma size between wildtype and *Phd2* hypomorphic animals (Fig 1A, 1C and 1D). Nonetheless, the numbers of regenerating cells were significantly enhanced in *Phd2* hypomorphic animals (Fig 1B). The tracking of myogenic markers during skeletal muscle regeneration revealed significant differences between the genotypes (Fig 2). We observed consistently earlier and higher peaks of Pax7, MyoD and myogenin in *Phd2*-hypomorphic mice. As myotrauma is likely to cause a hypoxic environment, we analysed the role of skeletal muscle HIF-1α and were able to immunohistochemically detect nuclear HIF-1α accumulation at all posttraumatic time points (Fig 3A). This is consistent with earlier studies showing HIF-1 in damaged myofibers [17] and damaged and regenerating muscle tissue [1]. Interestingly, we found a significantly increased percentage of regenerating muscle fibers in *Phd2*-hypomorphic mice expressing higher levels of HIF-1 (Fig 1B/1C). Immunohistochemical analysis of PHD2 revealed reduced numbers of positively stained myonuclei in regenerating areas of *Phd2* hypomorphic mice compared to wild type animals (Fig 3D).

With investigation of possible side effects of prolyl hydroxylase inhibitors (PIH), possible non-HIF hydroxylation targets of PHDs and factor inhibiting HIF (FIH) came into focus. In contrast to established mechanisms of non-HIF hydroxylation targets of FIH, like Ovarian tumor domain containing ubiquitin aldehyde binding protein 1 (OTUB1), non-HIF hydroxylation targets of PHDs are less well characterised. It is known that molecules of the NF-κB pathway and zinc fingers and homeoboxes 2 (ZHX2) are hydroxylated by PHDs but the exact mechanisms and especially a role in muscle regeneration are yet unknown [40]. Regarding the NF-κB pathway, we and others have shown that inhibition of PHDs (although this is mostly directed via PHD1 in the so far analysed settings) induces an activation of the classical NFκB pathway [41]. This would in turn result in an induction of *Hif-1a* mRNA and later also protein expression. This is why we assume that our observations are, at least in parts, mediated through HIF-1 due to *Phd2*-deficiency. Nonetheless, these are conclusions drawn by indirect observations as we did alter neither HIF1 function nor HIF1 activation directly in our experiments. Analysing the *Phd2* mRNA expression, it was surprisingly not significantly higher but even slightly lower in wild type animals 96 hours after trauma (Fig 3C). This puzzling effect might be explained by the fact that at this time point we detected high numbers of infiltrated cells (Fig 6A) which do not necessarily carry the *Phd2* hypomorphism to the same extend as skeletal muscle cells do. When we analysed the PHD2 protein expression via immunohistochemistry with a focus on regenerating muscle cells (that are targeted by the knockdown approach of this study; Fig 3E) it is significantly higher in wildtype animals compared to *Phd2* hypomorphic mice. This underlines our assumption that the discrepancy in Fig 3C can be explained with the abundance of various cell types in the traumatic area.

A classical HIF-target gene, VEGF, is expressed by myeloid cells [42] and myofibers [43]. VEGF-knockout mice showed no deterioration of skeletal muscle cell regeneration [1]. Interestingly, *Vegf* expression was not induced in ischemia-reperfusion injury and running training experiments in *Phd2*-hypomorphic mice [13, 44]. In contrast, we detected an induction of VEGF protein after myotrauma in immunohistochemical stainings of the traumatic areas (Fig 4A). In addition to this, *Vegf* mRNA expression in *Phd2*-hypomorphic mice after myotrauma was elevated at distinct time points, too (Fig 4D). It is conceivable that the type of damage impacts whether VEGF is induced. This study suggests that HIF-1-induced VEGF might function as an initiator of skeletal muscle regeneration six hours after myotrauma.

Classically, soluble growth factors such as HGF and IGF1 are responsible for satellite cell activation and myotrauma regeneration [19, 45, 46]. *Hgf* itself is described to be a HIF-1-regulated gene [47]. Interestingly, mRNA-expression of *Hgf* and *Igf1* was significantly more induced in *Phd2*-hypomorphic mice at 6 and 24 hours without showing any differences at later time points when skeletal muscle regeneration is active (Fig 4B/4C). This indicates a minor role of soluble growth factors in ongoing soft tissue trauma regeneration in this setting.

We investigated the degree of F4/80-positive macrophage accumulation at the injury site. Congruent with previous findings, we detected invading macrophages in the traumatic area [1]. However, macrophages from *Phd2*-hypomorphic mice reached the trauma site 24 hours earlier than in wild type mice (72h vs. 96h; Fig 6A). Thus, macrophage infiltration from *Phd2*-hypomorphic mice provides the mirror image of the situation with HIF-1-deficient macrophages from LysMCre/HIF-1a^+f/+f^ mice where reduced HIF-1 levels led to a significantly delayed infiltration compared to wild type mice [1].

Because of the critical role of macrophages and their HIF-dependent activation during skeletal trauma regeneration [1, 19, 30] we further focused on isolated bone marrow derived macrophages. BMDM from wild type and *Phd2*-hypomorphic mice did not show significant differences in the degree of expression of *Phd2* mRNA, indicating that the knockdown of *Phd2* might only mildly affect macrophages. Turning into consideration that a huge number of macrophages infiltrated the trauma tissue after 72 hours in Phd2-hypomorphic and 96 hours in wild type mice this is likely to influence mRNA inductions in whole muscle tissue from these time points onwards. FACS analysis of non-stimulated BMDM revealed a significantly higher expression of the anti-inflammatory marker CD206 in wild type macrophages pointing towards a predominantly proinflammatory phenotype of macrophages from *Phd2*-hypomorphic mice. This is in accordance with findings of Takeda *et al*., who identified HIF-1 to be more active in proinflammatory macrophages whereas HIF-2 was induced in macrophages polarised towards an anti-inflammatory phenotype [48]. Liu and co-workers showed a positive correlation between anti-inflammatory macrophages and the expression of myogenic markers in skeletal muscle regeneration [31]. We did not analyse the inflammatory profile of macrophages within trauma sites, but we observed macrophages in *Phd2*-hypomorphic animals earlier during trauma regeneration than in wild type mice. This earlier start of macrophage infiltration might be sufficient to initiate the myogenic program independent of the inflammatory status of the macrophages. A translational approach in skeletal muscle protection was done by Billin et al.: Inhibition of prolyl hydroxylases led to a skeletal muscle protective effect via HIF-mediated expression of iNOS in macrophages [49]. Because we did not isolate macrophages from traumatized muscles, we were not able to check for expression patterns of isolated macrophages; nevertheless, our work supports these previous findings.

Taking the so far discussed data into consideration, we see strong evidence that *Phd2* hypomorphic mice exhibit a more efficient recovery from hindlimb skeletal muscle trauma and that the observed effects are, at least in parts, HIF-mediated. The above mentioned myogenic markers Pax7, MyoD, and myogenin are not known to be direct HIF-1 targets so far. We found a significantly upregulated expression of Pax7 in *Phd2* hypomorphic mice 24 and 72 hours after trauma compared to wild type animals (Fig 2B). In line with this are *in vitro* findings showing an increased Pax7-expression under the influence of HIF-1 [26, 50, 51]. Inversely, a study reported that HIF-inhibition reduced myoblast differentiation [3, 52]. Herein, we provide evidence that stronger MyoD-expression is mediated via HIF-1 (Fig 2C), which supports recent findings by several groups [3, 53, 54] but contradicts a study that demonstrated MyoD degradation under hypoxia [55]. Considering a different pathophysiology in different methods of mechanical trauma, our findings support a promotion of satellite cell proliferation after mechanic muscle trauma which is regulated via HIF-1(α) [56]. Our results demonstrate that the higher regenerative potential and earlier trauma regeneration is associated with reduced PHD2 activity and higher HIF-1 expression (Fig 3A and 3B, respectively), which leads to an earlier and stronger activation of myogenic factors.

HIF and PHD dependency of CCR2 has not been described yet. CCR2 expression in regenerating muscle cells induces macrophage recruitment [57]. CCR2-knockout leads to reduced muscle regeneration, a prolonged inflammatory reaction and delayed angiogenesis [58, 59]. We detected a significantly higher *Ccr2* mRNA expression in *Phd2*-hypomorphic animals before and six hours after trauma (Fig 5D), suggesting a PHD2/HIF-1-dependency of *Ccr2*-expression. Although a direct correlation needs to be formally proven, the stronger induction found in *Phd2*-hypomorphic animals is subsequently followed by an earlier recruitment of macrophages, which is also HIF-dependent [60].

Glucose transporters (GLUT) are responsible for cellular glucose uptake. GLUTs are HIF-1 target genes [61]. *Phd2*-hypomorphic animals show a higher insulin sensitivity paired with lower basal insulin levels and a lower glycogen storage despite higher GLUT expression [13, 15]. Congruent with previous studies [62], we detected a higher expression of skeletal muscle *Glut*3 mRNA, indicating an enhanced glucose supply of the (trauma) tissue (Fig 7A). Muscle type phosphofructokinase (PFKM) is the prevalent isoform of this enzyme in skeletal muscles [63]. Elevated levels of PFKL (liver phosphofructokinase) have been described in the livers of *Phd2*-hypomorphic mice [13, 15]. Herein, we found that *Phd2*-hypomorphic mice showed a significantly higher expression of *Pfkm* mRNA under sham condition than wild type mice (Fig 7B), which would favour glycolysis and provide more energy supply in the regenerative phase. *Phd2*-hypomorphic mice of the sham group and 72 hours after myotrauma showed a significantly higher expression of monocarboxylate transporter (*Mct*) *3* (Fig 7C), which is required for the removal of glycolytic metabolites. Although these are findings are on mRNA expression level, they are likely to result in enhanced glucose and lactate metabolism. This metabolic switch to glycolysis may, although we did not check for metabolomics in serum samples, potentially contribute to the faster regeneration of skeletal muscle in *Phd2*-hypomorphic mice after trauma. Because many cytokines are not directly produced by mRNA-induction but are stored on a protein level, the mRNA expression analyses are, in parts, limited and should be expanded by protein analyses in following work.

In previous work, Scheerer *et al*. demonstrated that loss of HIF-1 in macrophages significantly delayed regeneration of skeletal muscle after soft tissue trauma whereas deletion of HIF-1α in muscle cells did not impair regeneration [1]. In this work, we were able to show that *Phd2*-deficency and HIF-1α accumulation provide an enhanced regeneration of skeletal muscle. This effect is not limited to either macrophages or muscle cells but might involve all cells with reduced *Phd2* activity. In view of the upcoming production of pharmacologic PHD inhibitors, application of PHD inhibitors may turn out to be advantageous after skeletal muscle trauma to accelerate regeneration [64, 65].

## Supporting information

S1 Fig mRNA-expression. qPCR analysis (whole muscle tissue) of selected parameters in myotrauma of traumatic and sham treated wild type (WT) and *Phd2*-hypomorphic (KD) mice. (A) hypoxia-inducible factor 1α; *Hif-1α*, (B) hypoxia-inducible factor 2α; *Hif-2α*, (C) prolyl hydroxylase 3; *Phd3*, (D) n-myc downstream regulated 1; *Ndrg1*, (E) phosphoglycerate kinase 1; *Pgk1*, (F) phosphoinositide-dependent kinase 1; *Pdk1*, (G) fibroblast growth factor; *Fgf*, (H) matrix metallopeptidase 9; *Mmp9* (mean ± SD; n 4–6; sham: n = 3). * P < 0.05, ** P < 0.01, *** P < 0.001.
(TIF)

S2 Fig Skeletal muscle repair is enhanced in *Phd2*-hypomorphic mice. Immunofluorescence staining of Pax7 in skeletal muscle of traumatic and sham treated wild type (WT) and *Phd2*-hypomorphic (KD) mice (time periods after trauma application are indicated). Stained are Pax7 (red), nuclei (DAPI; blue), intrinsic fluorescence of muscle (green). Shown are slides of 1 μm thickness (200x magnification; scale bar: 20 μm).
(TIF)

S3 Fig Skeletal muscle repair is enhanced in *Phd2*-hypomorphic mice. Immunofluorescence staining of MyoD in skeletal muscle of traumatic and sham treated wild type (WT) and *Phd2*-hypomorphic (KD) mice (time periods after trauma application are indicated). Stained are Pax7 (red), nuclei (DAPI; blue), intrinsic fluorescence of muscle (green). Shown are slides of 1 μm thickness (200x magnification; scale bar: 20 μm).
(TIF)

S4 Fig Skeletal muscle repair is enhanced in *Phd2*-hypomorphic mice. Immunofluorescence staining of Moygenin in skeletal muscle of traumatic and sham treated wild type (WT) and *Phd2*-hypomorphic (KD) mice (time periods after trauma application are indicated). Stained are Pax7 (red), nuclei (DAPI; blue), intrinsic fluorescence of muscle (green). Shown are slides of 1 μm thickness (200x magnification; scale bar: 20 μm).
(TIF)
