# Supplementary figures and images for: Prolyl hydroxylase domain 2 reduction enhances skeletal muscle tissue regeneration after soft tissue trauma in mice
Source: PLoS One. 2020 May 15;15(5):e0233261. doi: 10.1371/journal.pone.0233261 (PMC7228053; doi:10.1371/journal.pone.0233261)

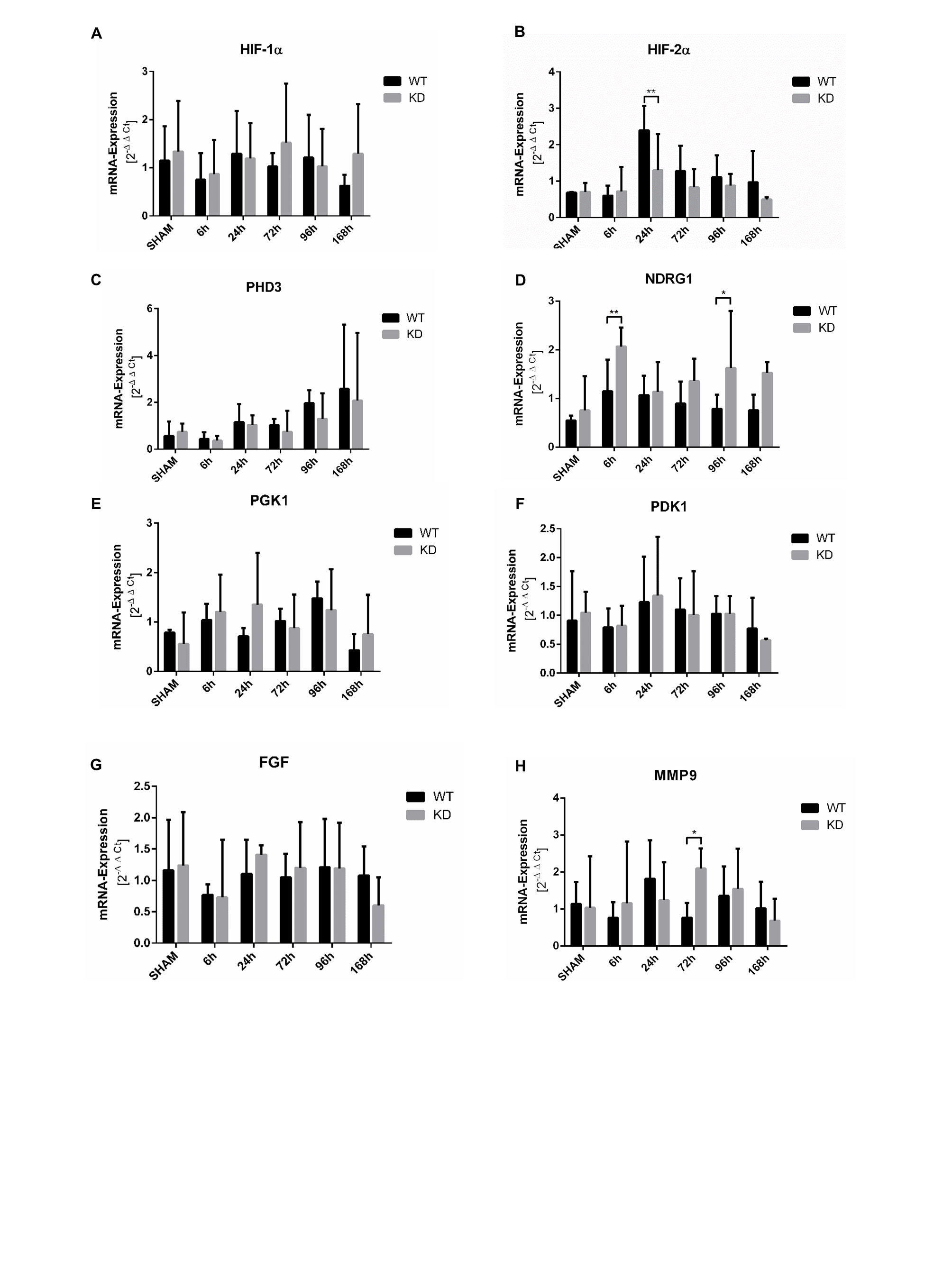

Supplement: S1 Fig — qPCR analysis (whole muscle tissue) of selected parameters in myotrauma of traumatic and sham treated wild type (WT) and Phd2-hypomorphic (KD) mice. (A) hypoxia-inducible factor 1α; Hif-1α, (B) hypoxia-inducible factor 2α; Hif-2α, (C) prolyl hydroxylase 3; Phd3, (D) n-myc downstream regulated 1; Ndrg1, (E) phosphoglycerate kinase 1; Pgk1, (F) phosphoinositide-dependent kinase 1; Pdk1, (G) fibroblast growth factor; Fgf, (H) matrix metallopeptidase 9; Mmp9 (mean ± SD; n 4–6; sham: n = 3). * P < 0.05, ** P < 0.01, *** P < 0.001. (TIF) [file pone.0233261.s001.TIF]

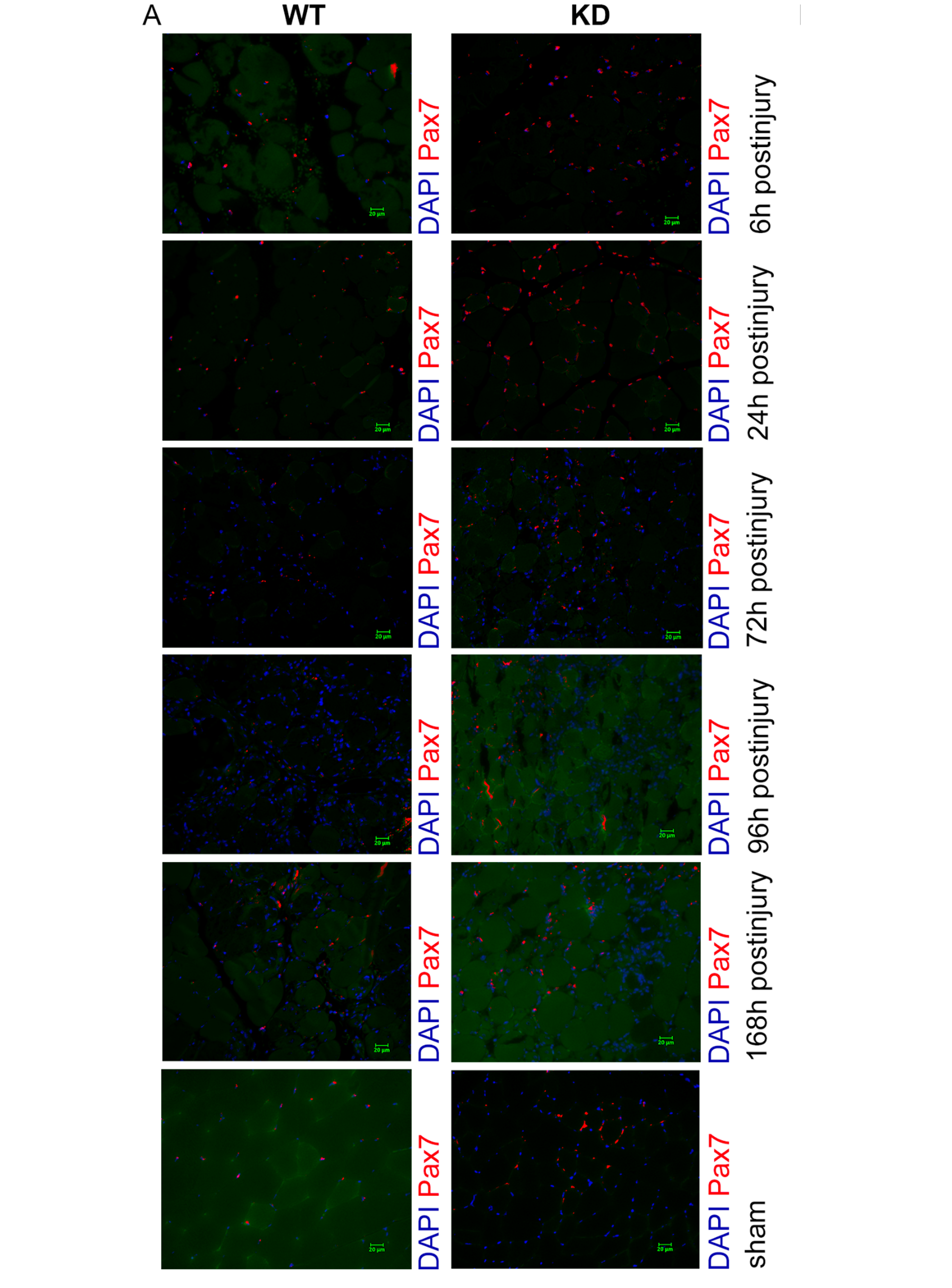

Supplement: S2 Fig — Immunofluorescence staining of Pax7 in skeletal muscle of traumatic and sham treated wild type (WT) and Phd2-hypomorphic (KD) mice (time periods after trauma application are indicated). Stained are Pax7 (red), nuclei (DAPI; blue), intrinsic fluorescence of muscle (green). Shown are slides of 1 μm thickness (200x magnification; scale bar: 20 μm). (TIF) [file pone.0233261.s002.TIF]

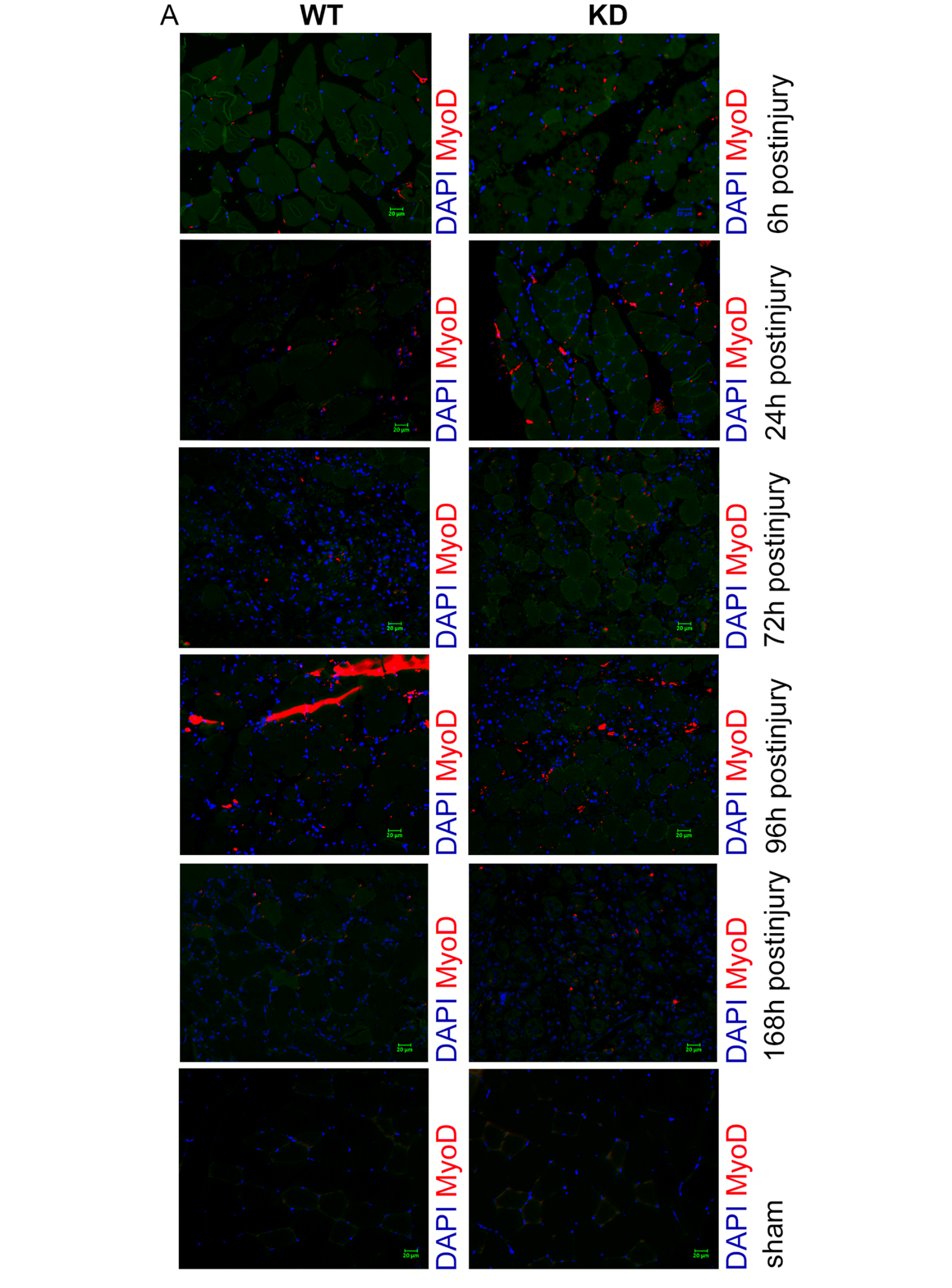

Supplement: S3 Fig — Immunofluorescence staining of MyoD in skeletal muscle of traumatic and sham treated wild type (WT) and Phd2-hypomorphic (KD) mice (time periods after trauma application are indicated). Stained are Pax7 (red), nuclei (DAPI; blue), intrinsic fluorescence of muscle (green). Shown are slides of 1 μm thickness (200x magnification; scale bar: 20 μm). (TIF) [file pone.0233261.s003.TIF]

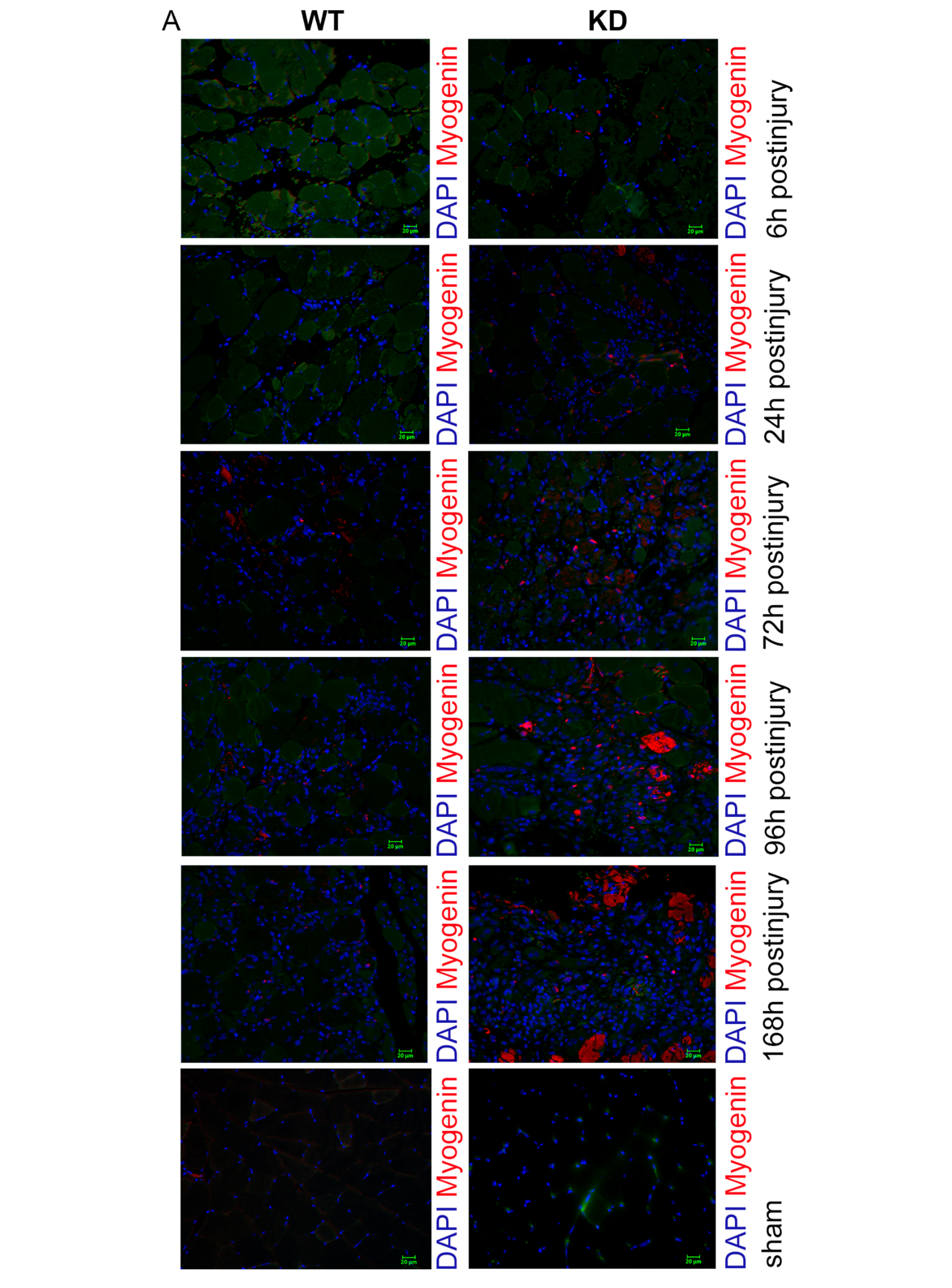

Supplement: S4 Fig — Immunofluorescence staining of Moygenin in skeletal muscle of traumatic and sham treated wild type (WT) and Phd2-hypomorphic (KD) mice (time periods after trauma application are indicated). Stained are Pax7 (red), nuclei (DAPI; blue), intrinsic fluorescence of muscle (green). Shown are slides of 1 μm thickness (200x magnification; scale bar: 20 μm). (TIF) [file pone.0233261.s004.TIF]
